# Importin-7-dependent nuclear translocation of the *Flavivirus* core protein is required for infectious virus production

**DOI:** 10.1371/journal.ppat.1012409

**Published:** 2024-08-15

**Authors:** Yumi Itoh, Yoichi Miyamoto, Makoto Tokunaga, Tatsuya Suzuki, Akira Takada, Akinori Ninomiya, Tomomi Hishinuma, Mami Matsuda, Yoshihiro Yoneda, Masahiro Oka, Ryosuke Suzuki, Yoshiharu Matsuura, Toru Okamoto

**Affiliations:** 1 Department of Microbiology, Juntendo University School of Medicine, Tokyo, Japan; 2 Laboratory of Nuclear Transport Dynamics, National Institutes of Biomedical Innovation, Health and Nutrition, Osaka, Japan; 3 Laboratory of Biofunctional Molecular Medicine, National Institutes of Biomedical Innovation, Health and Nutrition, Osaka, Japan; 4 Institute for Advanced Co-Creation Studies, Research Institute for Microbial Diseases, Osaka University, Osaka, Japan; 5 Central Instrumentation Laboratory, Research Institute for Microbial Diseases, Osaka University, Osaka, Japan; 6 Department of Virology II, National Institute of Infectious Diseases, Tokyo, Japan; 7 The Research Foundation for Microbial Diseases of Osaka University, Osaka, Japan; 8 Laboratory of Virus Control, Center for Infectious Disease Education and Research, Osaka University, Osaka, Japan; 9 Research Institute for Microbial Diseases, Osaka University, Osaka, Japan; National Institute of Allergy and Infectious Diseases, UNITED STATES OF AMERICA

## Abstract

*Flaviviridae* is a family of positive-stranded RNA viruses, including human pathogens, such as Japanese encephalitis virus (JEV), dengue virus (DENV), Zika virus (ZIKV), and West Nile virus (WNV). Nuclear localization of the viral core protein is conserved among *Flaviviridae*, and this feature may be targeted for developing broad-ranging anti-flavivirus drugs. However, the mechanism of core protein translocation to the nucleus and the importance of nuclear translocation in the viral life cycle remain unknown. We aimed to identify the molecular mechanism underlying core protein nuclear translocation. We identified importin-7 (IPO7), an importin-β family protein, as a nuclear carrier for *Flaviviridae* core proteins. Nuclear import assays revealed that core protein was transported into the nucleus via IPO7, whereas *IPO7* deletion by CRISPR/Cas9 impaired their nuclear translocation. To understand the importance of core protein nuclear translocation, we evaluated the production of infectious virus or single-round-infectious-particles in wild-type or IPO7-deficient cells; both processes were significantly impaired in IPO7-deficient cells, whereas intracellular infectious virus levels were equivalent in wild-type and IPO7-deficient cells. These results suggest that IPO7-mediated nuclear translocation of core proteins is involved in the release of infectious virus particles of flaviviruses.

## Author summary

The Flaviviridae family, which includes Japanese encephalitis virus (JEV), dengue virus (DENV), Zika virus (ZIKV), and West Nile virus (WNV), possesses a single-stranded positive-sense RNA and conducts viral RNA replication, translation, and particle formation in the endoplasmic reticulum. The core proteins of flaviviruses, which form viral capsid, have been recognized to localize in not only the cytoplasm but also the nucleus, particularly in the nucleolus. Although this nuclear translocation of the core proteins is conserved within the Flaviviridae family, the underlying molecular mechanisms and their contribution to the viral life cycle remain largely unknown. Our *in vitro* biochemical assay and mass spectrometry analysis identified IPO7 as a nuclear carrier for Flaviviridae core proteins, such as those in JEV, DENV, ZIKV, and WNV. Moreover, we found that IPO7-mediated nuclear translocation of core proteins is important for the release of infectious viral particles. Our data suggest that the Flaviviridae family evolved to use the IPO7-mediated translocation of core proteins for efficient viral production. Therefore, IPO7 might be a target for the development of antiviral drugs against a broad range of Flaviviruses.

## Introduction

Viruses belonging to the family *Flaviviridae*, including the genus *Flavivirus*, comprising Japanese encephalitis virus (JEV), dengue virus (DENV), Zika virus (ZIKV), West Nile virus (WNV), and tick-borne encephalitis virus, which are arthropod-borne human pathogens [1,2]. Infection with JEV or WNV can cause fatal neurological diseases in humans [3,4]. ZIKV is a neurotropic virus associated with Guillain–Barré syndrome, neuropathy, and myelitis in adults and children [5,6]. ZIKV infection during pregnancy can cause microcephaly in infants. DENV infection can cause dengue fever and dengue hemorrhagic fever [7]. The risk of infectious diseases caused by flaviviruses is increasing globally. DENV causes an estimated 390 million infections and 100 million symptomatic cases per year. Moreover, the number of DENV infections and DENV-related deaths has been increasing in the last two decades [8]. The highest number of dengue-related deaths was observed in 2022 in Bangladesh, compared to the previous years [9]. Approximately 70,000 cases of JEV infection are reported annually [10]. However, owing to the lack of cure for diseases caused by flavivirus infections, only severe symptoms are relieved [11]. Therefore, prophylactic and therapeutic measures, including vaccines, are urgently needed.

*Flaviviridae* are single-stranded positive-sense RNA viruses. Viral RNA is released from viral particles and is directly translated into a polyprotein of approximately 3,000 amino acids. The polyprotein is cleaved into 10 viral proteins by host or viral proteases. Flaviviridae RNA replication occurs in so-called viral replication factories, double-membrane vesicles, that are partly derived from the endoplasmic reticulum (ER), and viral particle production occurs in ER. Therefore, for many years, the viral life cycle was considered to be completed in the cytoplasm. However, a non-structural protein, NS5, is localized in the nucleus [12]. Some flavivirus core proteins are translocated from the cytoplasm into the nucleus [13]. We have demonstrated that Ala substitution in the JEV core protein at Gly42 and Pro43 impaired the nuclear translocation of the core protein [14], and recombinant JEV possessing a core protein lacking the nuclear translocation signal showed impaired propagation and attenuation in a mouse model. Mutations in the JEV core protein impaired the formation of viral infectious particles [15]. However, mutations of the core protein may affect the three-dimensional structure of the protein and have unknown side effects in addition to affecting nuclear localization. We recently showed that core protein nuclear translocation is conserved among *Flaviviridae* and may be a target for broad-ranging anti-flavivirus drugs [16]; however, how the core protein is translocated from the cytoplasm to the nucleus is unclear.

Macromolecular transport between the cytoplasm and nucleus via the nuclear pore complex (NPC) is a fundamental process for information delivery for appropriate gene expression tightly regulated by importin/karyopherin family proteins [17]. The importin/karyopherin family consists of more than 20 proteins that are categorized into two groups: importin-α and importin-β. Importin-α proteins are classical nuclear localization signal (cNLS) receptors and function as adaptor molecules linking cNLS-containing proteins with importin-β1, an importin-β family protein [18,19]. In the cytoplasm, cNLS-containing proteins are recognized by importin-α and form a trimeric complex with importin-β1. The complex passes through the nuclear pore via interaction of importin-β1 with components of the NPC. In the nucleus, the GTP-bound form of Ran (RanGTP), a guanine nucleotide-binding protein that is abundant in the nucleus, strongly associates with importin-β1 to release the cNLS-containing protein. Approximately 20 importin-β family members have been identified and function in nuclear import, export, or bidirectional transport [20,21]. For nuclear import, importin-β proteins such as transportin directly bind to specific cargo proteins without importin-α, and the complex passes through the NPC. Cargos are released from importin-β proteins by binding with RanGTP in the nucleus.

In this study, we aimed to elucidate the molecular mechanism underlying the nuclear translocation of core proteins of flaviviruses. Specifically, we investigated the involvement of importin-7 (IPO7) in the nuclear transport of core proteins as well as the role of core protein nuclear translocation in the flavivirus life cycle.

## Results

### Importin-β-dependent nuclear translocation of flavivirus core proteins

**Fig 1:**
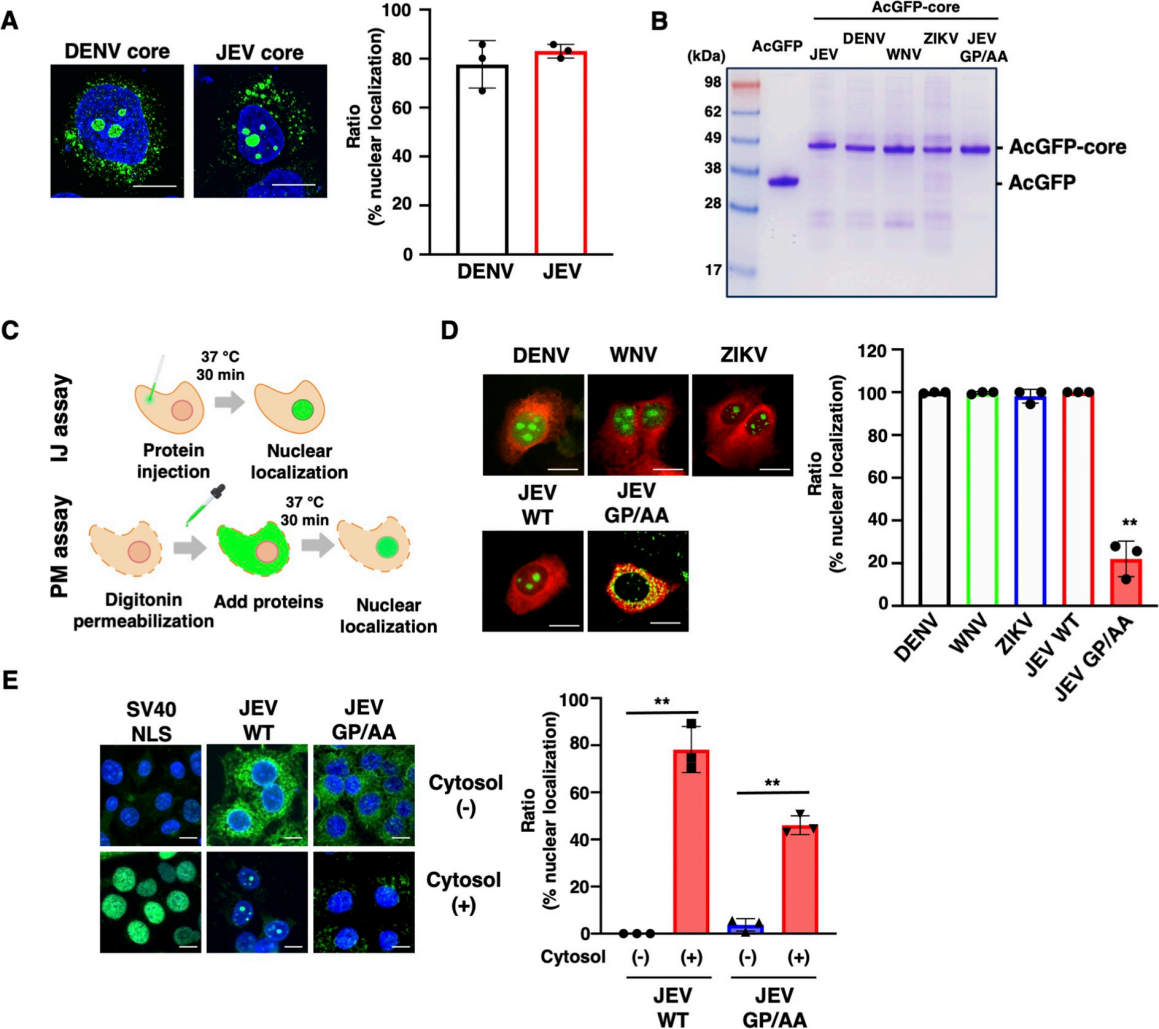
*In vitro* biochemical analysis of the nuclear localization of flavivirus core proteins. **(A)** The plasmid coding non-tagged JEV core or DENV core was transfected in Huh7 cells. After two days, transfectants were fixed and stained with the anti-JEV core or anti-DENV core antibody. The scale bar indicates 20 μm. The right graph shows the quantification of the ratio of nuclear translocation. **(B)** Recombinant AcGFP-core of each virus was expressed in the baculovirus expression system and visualized by SDS-PAGE and CBB stain. **(C)** Experimental design for the analysis of the nuclear localization of core proteins. In the injection (IJ) assay, recombinant core proteins and immunoglobulin (IgG) were injected into Huh7 cells. After 30 min, the localization of recombinant core proteins was observed through confocal laser microscopy. In the permeabilization (PM) assay, Huh7 cells were treated with digitonin, and the permeabilized cells were then incubated with recombinant core proteins. After 30 min, the localization of recombinant core proteins was observed through confocal laser microscopy. **(D)** Localization of core proteins of flaviviruses by the IJ assay. Recombinant AcGFP-core protein and IgG labeled with Alexa Fluor 555 were injected into Huh7 cells. The scale bar indicates 20 μm. The right graph is the quantification of the ratio of nuclear translocation. **(E)** PM assay using GST and GFP fused with the NLS of SV40 large T antigen (SV40 NLS) or AcGFP-JEV core WT or AcGFP-JEV core GP/AA. Rabbit reticulocyte lysate was employed as a cytosol source including import factors. The scale bar indicates 20 μm. The right graph is the quantification of the ratio of nuclear translocation. All data are representative of three independent experiments. Significance (*p < 0.05; **p < 0.01; n. s., not significant) was determined using Student’s t-test (n = 3). Created with Biorender.com.

The core proteins of JEV, DENV, ZIKV, and WNV are translocated in the cytoplasm and nucleolus [14,16]. Consistent with the previous finding, the plasmid transfection of non-tagged JEV core or DENV core protein showed that 80% of both core proteins was translocated in the nucleus (Fig 1A). In the present study, we used two different *in vitro* nuclear transport assays using recombinant core proteins. Green fluorescent protein (AcGFP)-fused recombinant core proteins were generated using baculovirus-insect expression systems. Coomassie brilliant blue (CBB) staining and western blotting (WB) analysis showed that all recombinant proteins were of high purity with no cleavage products (Figs 1B and S1). First, AcGFP-fused recombinant core proteins, with Alexa Fluor 555-conjugated antibodies as an injection marker, were injected into the cytoplasm of Huh7 cells using capillary needles. After the cells were incubated for 30 min, nuclear localization of the core proteins was observed based on GFP intensity (injection [IJ] assay). Second, digitonin-permeabilized Huh7 cells were incubated with GFP-fused core proteins and rabbit reticulocyte lysates. Nuclear translocation of the core proteins was observed through microscopy (permeabilization [PM] assay) (Fig 1C). The IJ assay revealed that GFP-fused core proteins of JEV, DENV, ZIKV, and WNV translocated from the cytoplasm to the nucleus. In contrast, Ala substitution at Gly42 and Pro43 (GP) of the JEV core protein (GP/AA) abolished its nuclear translocation (Fig 1D). In the PM assay, GST- and GFP-fused recombinant protein harboring a Simian virus-40 NLS sequence (SV40 NLS) and wild-type (WT) JEV core protein clearly translocated in the nucleus; however, the GP/AA JEV core mutant showed no nuclear translocation (Fig 1E). Compared to the plasmid transfection of non-tagged core protein, the majority of recombinant AcGFP-core was translocated in the nucleus. This can explain the effect of AcGFP on ER retention of core proteins.

**Fig 2:**
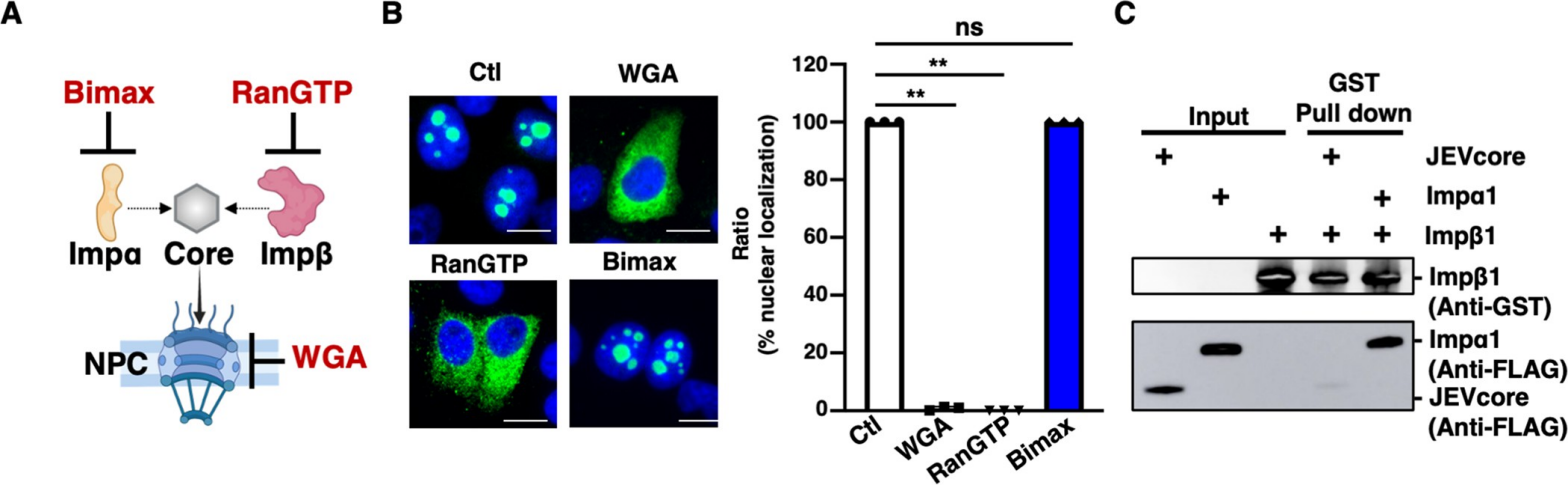
Nuclear pore complex (NPC)-mediated nuclear localization of core proteins is importin-α-independent and importin-β-dependent. **(A)** Scheme of the experiment used to assess the involvement of importin-α (Impα), importin-β (Impβ), and NPC. **(B)** The injection (IJ) assay was used to assess the involvement of importin-α, importin-β, and NPC in core protein nuclear localization. Bimax, RanGTP, and WGA were used as inhibitors of importin-α, importin-β, and nuclear pore complexes, respectively. The scale bar indicates 20 μm. The right graph is the quantification of the ratio of nuclear translocation. **(C)** A GST pull-down assay was performed using GST-fused importin-β1 mixed with flag-tagged core protein or importin-α1, respectively. Input and immunoprecipitant samples were subjected to SDS-PAGE and detected by western blotting using anti-GST and anti-FLAG antibodies. All data are representative of three independent experiments. Significance (*p < 0.05; **p < 0.01; n. s., not significant) was determined using Student’s t-test (n = 3). Created with Biorender.com.

To understand the molecular pathway underlying nuclear transport of the core protein, we used several inhibitors. Bimax strongly binds to importin-α to inhibit importin-α-dependent nuclear translocation [22,23]. Wheat germ agglutinin (WGA), an N-acetylglucosamine-binding lectin, inhibits active nuclear transport by binding to nucleoporins, which are heavily O-N-acetylglucosamine-modified [24]. A dominant-negative Ran mutant mutated at Gln69 to Leu (RanGTP Q69L) and defective in GTP hydrolysis strongly binds to importin-β family proteins to inhibit importin-dependent nuclear translocation [25,26] (Fig 2A). We performed the IJ assay using the JEV core protein mixed with Bimax, Q69LRanGTP, or WGA. Injection with WGA inhibited core protein nuclear translocation, indicating that it migrates into the nucleus via the NPC (Fig 2B). While Bimax showed no effect on core protein nuclear translocation, RanGTP Q69L clearly retained the core protein in the cytoplasm (Fig 2B). This indicates that an importin-β-dependent pathway underlies the nuclear transport of the JEV core protein. As importin-β1 is a well-known nuclear transporter within the family, we first examined whether importin-β1 can directly bind to the core protein independent of importin-α. A glutathione S-transferase (GST) pull-down assay revealed that GST-importin-β1 did not interact with flag-tagged JEV core protein under the condition in which it bound to importin-α1 (Fig 2C). These results suggest that the JEV core protein is transported into the nucleus in an importin-β family-dependent manner but not via importin-β1.

### Identification of IPO7 as a carrier for core protein nuclear localization

**Fig 3:**
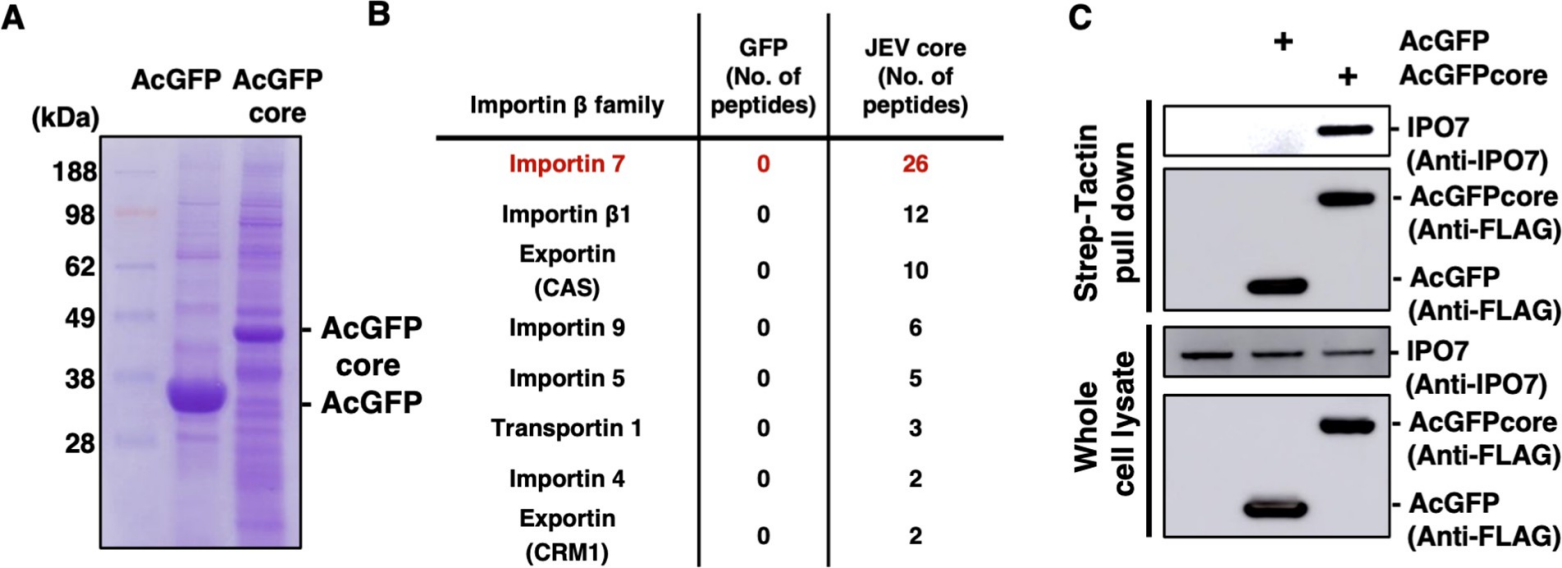
Importin-7 (IPO7) specifically interacts with the Japanese encephalitis virus (JEV) core protein. **(A)** AcGFP-FOS or AcGFP-JEV core-FOS was transfected into 293T cells and precipitated using Strep-Tactin Sepharose beads. The pull-down samples were analyzed through mass spectrometry (MS). **(B)** List of importin-β family proteins identified by MS analysis. The numbers of unique peptides identified by MS are indicated. **(C)** AcGFP-FOS or AcGFP-JEV core-FOS was transfected into 293T cells and precipitated using Strep-Tactin Sepharose beads. The precipitated samples were subjected to SDS-PAGE and detected by western blotting using anti-IPO7 and anti-flag antibodies. The data are representative of three independent experiments.

We next attempted to identify proteins binding to the JEV core protein through mass spectrometry (MS) as previously reported [27,28]. Strep tag-fused AcGFP or AcGFP-JEV core protein was expressed in 293T cells and immunoprecipitated using Strep-Tactin beads (Fig 3A). Among the core-binding proteins, we selected proteins belonging to the importin-β family, as shown in Fig 3B, and focused on IPO7 because it was identified as the most abundant peptides, and no direct interaction with Impotin-β1 was confirmed in Fig 2C. To validate the interaction between IPO7 and the JEV core protein, we used an immunoprecipitation assay; IPO7 was pulled down with the AcGFP-JEV core protein using an anti-flag antibody (Fig 3C).

**Fig 4:**
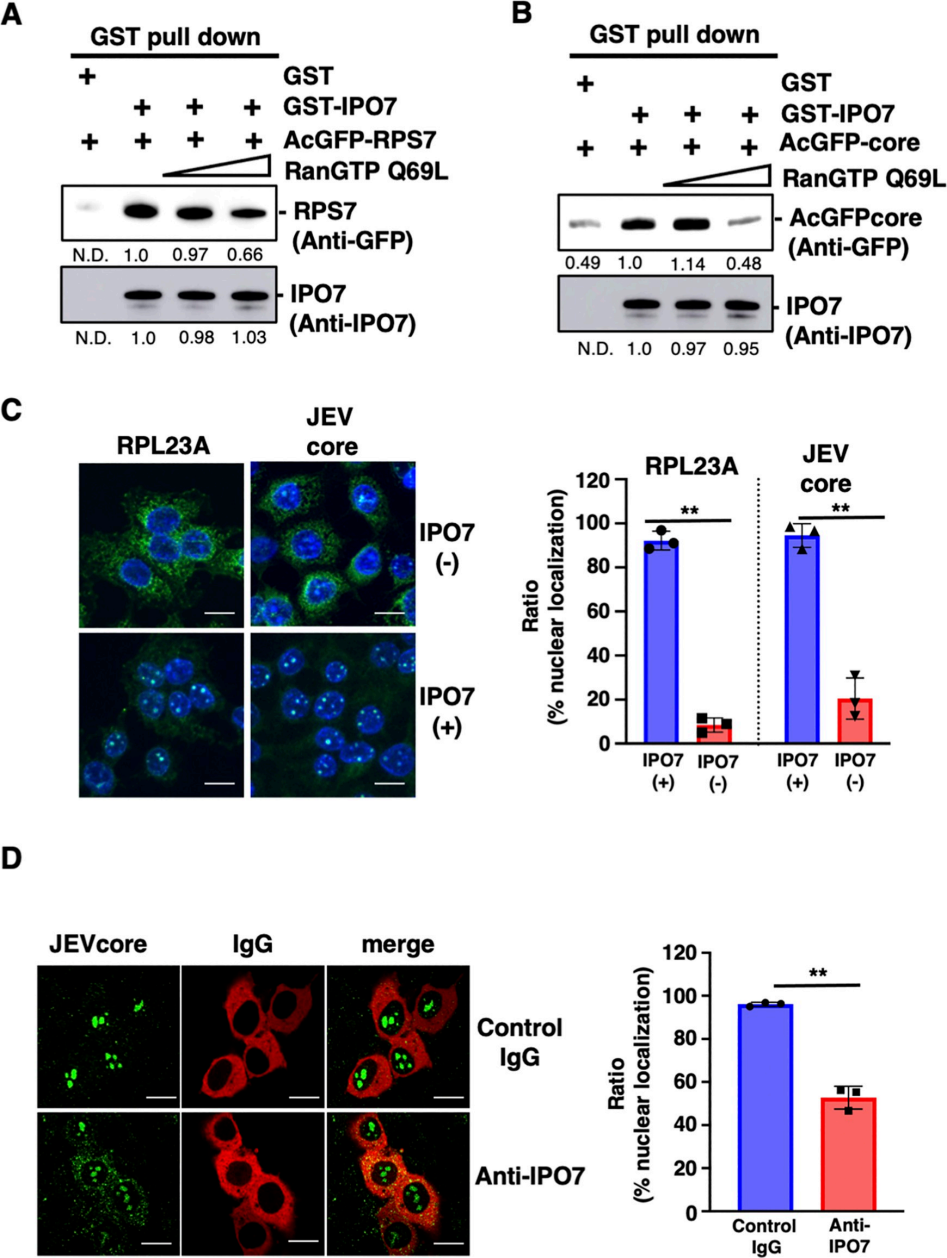
Importin-7 (IPO7) can transport flavivirus core proteins into the nucleus. **(A)** GST or GST-IPO7 was incubated with AcGFP-RPS7 in the presence or absence of 100 pmol or 770 pmol RanGTP Q69L. The pulled-down proteins were subjected to SDS-PAGE and detected by western blotting using anti-IPO7 and anti-GFP antibodies. The numbers indicate amounts of each protein calculated using the Image J software. **(B)** Either GST or GST-IPO7 was incubated with AcGFP-JEV core with or without 100 pmol or 770 pmol RanGTP Q69L. The pulled-down proteins were subjected to SDS-PAGE and detected by western blotting using anti-IPO7 and anti-GFP antibodies. The numbers indicate amounts of each protein calculated using the Image J software. **(C)** The permeabilization (PM) assay was performed using recombinant IPO7 protein. Digitonin-permeabilized Huh7 cells were incubated with GST-GFP-RPL23A or AcGFP-JEV core proteins with or without IPO7. Localization of core protein was observed through confocal laser microscopy. The scale bar indicates 20 μm. The right graph is the quantification of the ratio of nuclear translocation. **(D)** IPO7 antibody or control IgG was injected with recombinant Japanese encephalitis virus (JEV) core protein and IgG conjugated with Alexa Fluor 594. Core protein localization was observed through confocal laser microscopy. The scale bar indicates 20 μm. The right graph is the quantification of the ratio of nuclear translocation. All data are representative of three independent experiments. Significance (*p < 0.05; **p < 0.01; n. s., not significant) was determined using Student’s t-test (n = 3).

To assess whether IPO7 binds directly to the core protein and then transports it into the nucleus, we purified bacterially expressed recombinant proteins and performed pull-down assays. As a positive control, we used ribosomal protein S7 (RPS7), which has been shown to be a specific cargo of IPO7, similar to RPL23A [29]. As shown in Fig 4A, AcGFP-RPS7 was pulled down with GST-IPO7 protein. The direct interaction between the importin-β family and its substrates is disrupted by QanGTP R69L [25,26]. To confirm whether IPO7 binds to RPS7 or JEV core protein in a manner similar to the importin-β family and its substrates, RanGTP Q69L was added to the GST pull-down assay. The binding between IPO7 and RPS7 was disrupted by the addition of RanGTP Q69L (Fig 4A). We identified a direct interaction between IPO7 and the JEV core protein, and the addition of RanGTP more effectively dissociated this binding than that between IPO7 and RPS7 (Fig 4B). These results suggest that the binding mode of IPO7 to the JEV core protein is similar to that to RPS7.

Finally, we examined whether IPO7 can transport the JEV core protein into the nucleus. PM assay showed that the JEV core protein was translocated into the nucleus when IPO7 recombinant protein was added, which aligns with the findings for RPL23A (Fig 4C). Microinjection of an antibody specific for IPO7 together with the JEV core protein into the cytoplasm significantly inhibited the nuclear translocation of the JEV core protein (Fig 4D). These results showed that IPO7 acts as a nuclear carrier for the JEV core protein.

### IPO7 is a conserved nuclear carrier of flavivirus core proteins

**Fig 5:**
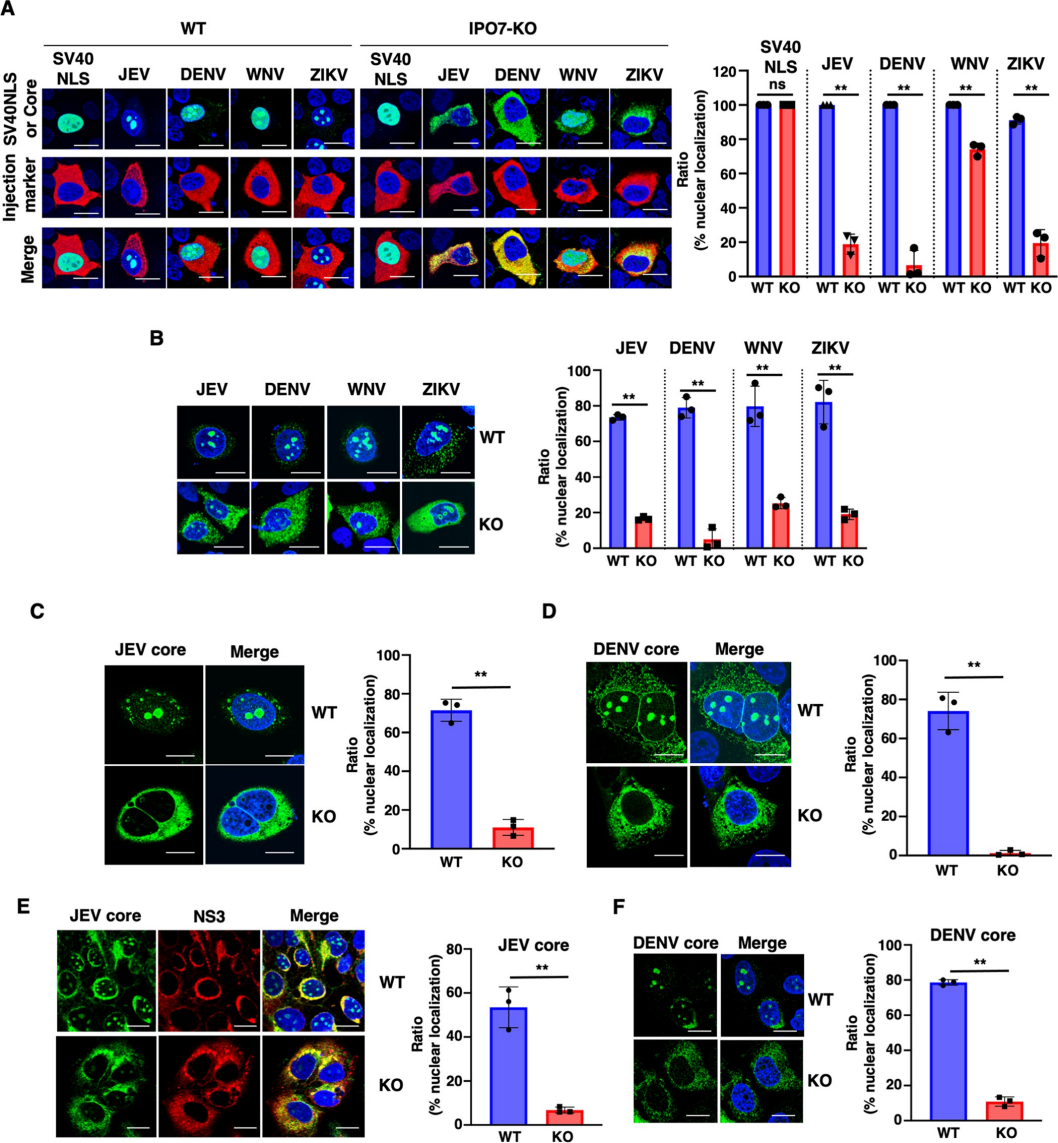
IPO7 deficiency in Huh7 cells impairs the nuclear translocation of core proteins of *Flaviviridae*. **(A)** GST and GFP fused the SV40 large T antigen NLS, or AcGFP fused core proteins of Flaviviridae were microinjected into the cytoplasm of WT or KO cells with red fluorescence protein-conjugated antibody as a cytoplasmic injection marker. The scale bar indicates 20 μm. The right graph is the quantification of the ratio of nuclear translocation. **(B)** Plasmids encoding AcGFP fused with core proteins of *Flaviviridae* were transfected into wild-type (WT) or IPO7-KO Huh7 cells. The scale bar indicates 20 μm. The right graph is the quantification of the ratio of nuclear translocation. **(C, D)** Plasmids encoding non-tagged JEV or DENV core proteins were transfected into WT or IPO7-KO Huh7 cells. The scale bar indicates 20 μm. The right graph is the quantification of the ratio of nuclear translocation. **(E, F)** The WT or IPO7-KO Huh7 cells were infected with JEV and DENV. Subcellular localization of core protein was observed using indicated antibodies. The scale bar indicates 20 μm. The right graph is the quantification of the ratio of nuclear translocation. All data are representative of three independent experiments. Significance (*p < 0.05; **p < 0.01; n. s., not significant) was determined using Student’s t-test (n = 3).

We generated IPO7-knockout (KO) cells using the CRISPR/Cas9 technology (S2A Fig). To examine the effect of IPO7 on the nuclear translocation of core proteins of flaviviruses, recombinant core proteins of JEV, DENV, WNV, or ZIKV or SV40-NLS was injected into the cytoplasm of WT or IPO7-KO Huh7 cells. IJ assays showed that SV40-NLS was translocated into the nucleus in both WT and IPO7-KO Huh7 cells. The core proteins of JEV, DENV, and ZIKV were retained in the cytoplasm in IPO7-KO Huh7 cells but not in WT Huh7 cells (Fig 5A), while the core protein of WNV was partially retained in the cytoplasm in IPO7-KO Huh7 cells. Consistent with this finding, AcGFP-core protein plasmid transfection showed that nuclear translocation of the JEV, DENV, WNV, and ZIKV core proteins was partially inhibited in IPO7-KO Huh7 cells but not in WT Huh7 cells (Fig 5B). We generated several IPO7-KO Huh7 cell clones (S2B Fig). AcGFP-core protein plasmid transfection showed that nuclear translocation of the AcGFP-JEV core protein was partially inhibited in IPO7-KO Huh7 cells but not in WT Huh7 cells (S2C Fig). Plasmid transfection of non-tagged JEV or DENV core protein showed that nuclear translocation of the core proteins was inhibited in IPO7-KO Huh7 cells but not in WT Huh7 cells (Fig 5C and 5D). To examine whether other viral proteins might be involved in IPO7-mediated nuclear transport, DENV NS5 plasmid transfection or DENV infection was performed in WT and IPO7-KO Huh7 cells. No difference was observed in the nuclear localization of NS5 in both cells (S2D and S2E Fig). Finally, we examined core protein localization in JEV or DENV-infected IPO7-KO Huh7 cells. Nuclear translocation of JEV and DENV core proteins was observed in WT Huh7 cells but not in IPO7-KO Huh7 cells (Fig 5E and 5F), suggesting that IPO7 is a conserved nuclear importer of the core proteins of *Flaviviridae*.

### IPO7-mediated nuclear translocation of core protein in viral life cycles

**Fig 6:**
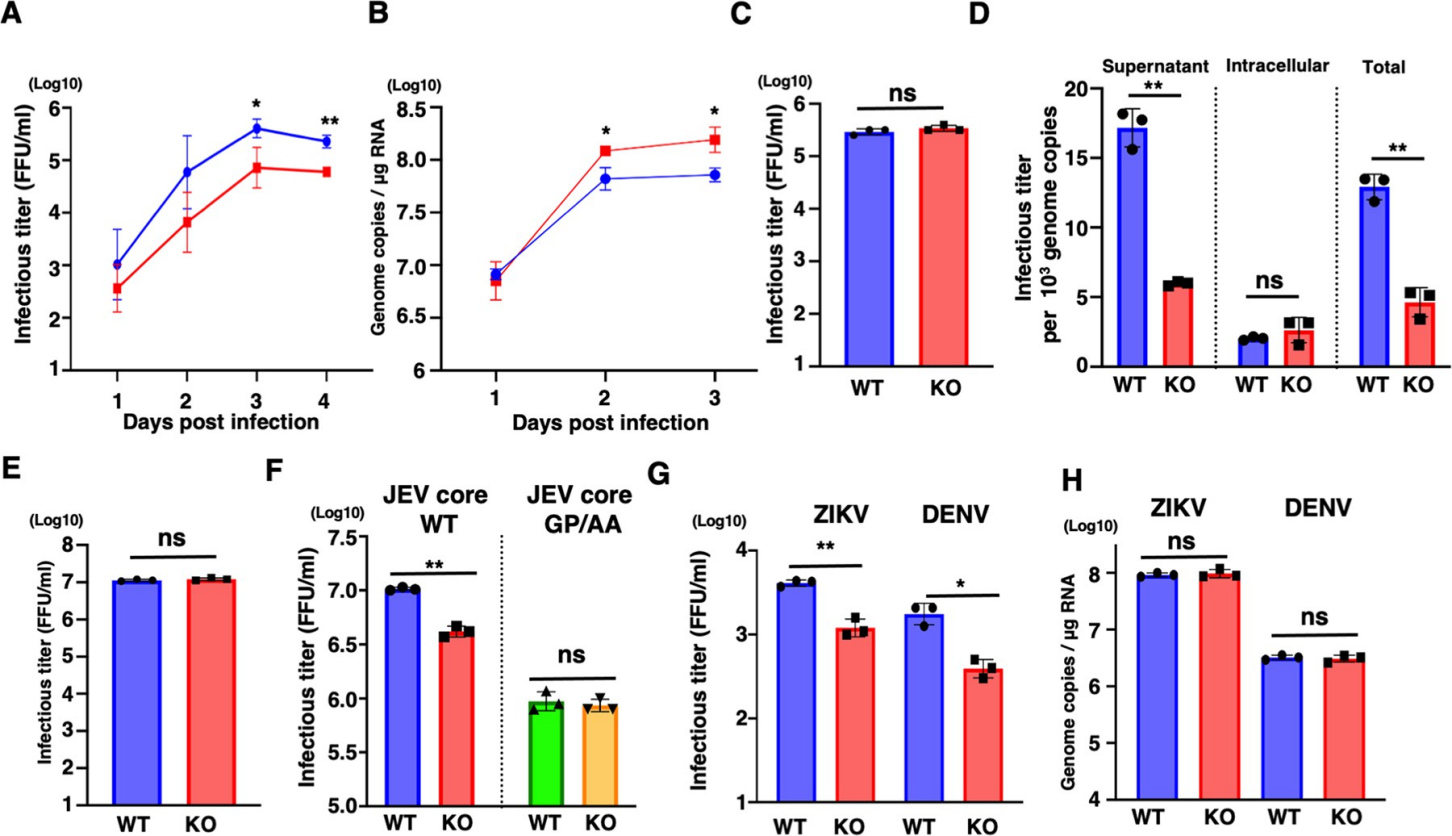
IPO7 affects infectious viral release in the viral life cycle. **(A, B)** WT or IPO7-KO Huh7 cells were infected with JEV at a multiplicity of infection (MOI) of 5. The viral titers in supernatants **(A)** and intracellular viral RNA **(B)** were quantified at each time point. **(C)** WT or IPO7-KO Huh7 cells infected with JEV at an MOI of 5 were collected three days post-infection. Intracellular viral titers were determined after each of repeated freeze–thaw cycles. **(D)** WT or IPO7-KO Huh7 cells were infected with JEV. Viral titers were determined for each virus. WT Huh7 cells were infected with the virus at a multiplicity of infection (MOI) of 5. After 2 days, the supernatant was collected, and the viral titer was determined. **(E)** WT or IPO7-KO Huh7 cells were infected with JEV at an MOI of 5. The supernatants were collected at 2 days post infection. Each viral supernatant was infected into Huh7 cells at MOI of 5. After 2 days, each supernatant was collected and was determined viral titer. **(F)** WT JEV or mutant JEV harboring GP/AA core was infected at an MOI of 1 into WT or IPO7-KO Huh7 cells. The supernatants were collected at 2 days post infection and determined the viral titers. **(G, H)** WT or IPO7-KO Huh7 cells were infected with ZIKV and DENV at a multiplicity of infection (MOI) of 5. The viral titers in supernatants **(G)** and intracellular viral RNA **(H)** at three days post-infection were quantified. All data are representative of three independent experiments. Significance (*p < 0.05; **p < 0.01; n.s., not significant) was determined using Student’s *t*-test (*n* = 3).

We then examined the role of IPO7-dependent nuclear translocation of core protein on the viral life cycle. The viral titer produced from IPO7-KO Huh7 cells was impaired compared to that from WT cells (Fig 6A). Cytotoxicity of JEV infection showed no difference in WT or IPO7-KO Huh7 cells (S2F Fig). In contrast, intracellular viral RNA was upregulated in IPO7-KO Huh7 cells (Fig 6B). We quantified intracellular infectious virus particles following repeated freezing and thawing of virus-infected cells. Intracellular infectious virus levels were equivalent in WT or IPO7-KO Huh7 cells (Fig 6C). To clarify the population of infectious virus and viral genome, supernatants and virus-infected cells were collected, and viral titers and copy numbers of the viral genome were determined. Although the population of infectious virus in supernatant was impaired in IPO7-KO Huh7 cells, no significant differences were observed in the intracellular levels (Fig 6D). Subsequently, equal amounts of infectious JEV particles collected from WT or IPO7-KO Huh7 cells were infected into WT Huh7 cells. No difference was observed in infectivity between viruses produced by WT and IPO7-KO Huh7 cells (Fig 6E). Subsequently, we generated recombinant JEV with WT or GP/AA core and infected them with WT and IPO7-KO Huh7 cells. Although viral titer of recombinant JEV possessing WT core was impaired in IPO7-KO Huh7 cells, that of recombinant JEV possessing GP/AA core showed no difference in WT and IPO7-KO Huh7 cells (Fig 6F). To examine our results, WT and IPO7-KO Huh7 cells were infected with ZIKV or DENV. Consistent with the observation on JEV, the viral titer of ZIKV or DENV was impaired in IPO7-KO Huh7 cells (Fig 6G). In contrast to JEV, intracellular ZIKV or DENV RNA showed no difference in IPO7-KO Huh7 cells (Fig 6H). Other IPO7-KO Huh7 cells impaired the production of JEV (S3A Fig). Finally, to examine the possibility that our results were dependent on Huh7 cells, we generated IPO7-KO HEK293T cells and showed that the viral titer of JEV was impaired in IPO7-KO HEK293T cells (S3B and S3C Fig). These results suggest that IPO7 is crucial in the life cycle of these viruses.

### IPO7 is involved in the release of infectious virus particles of *Flaviviridae*

**Fig 7:**
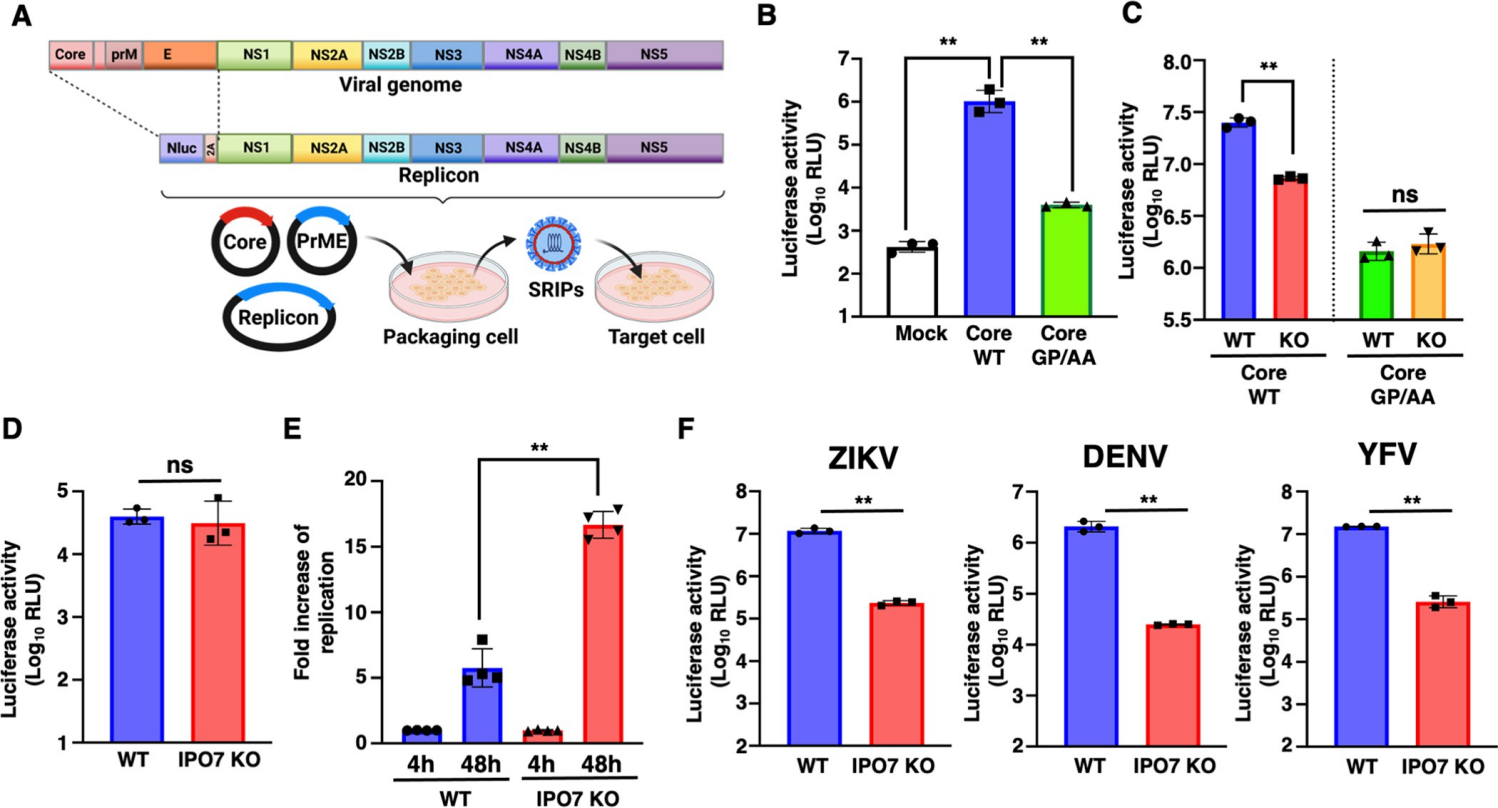
Disruption of the nuclear localization of the core proteins of *Flaviviridae* impairs efficient infectious virus release. **(A)** Schematic procedure of the single-round infectious particle (SRIP) generation experiments. **(B)** Three different plasmids containing the core, prME, and replicon were transfected into 293T cells to produce SRIPs. SRIPs produced by wild-type (WT) or GP/AA core protein were used to infect Vero cells. Nluc reporter activity was quantified using the Nano-Glo Luciferase Assay System. **(C)** SRIPs harboring WT or GP/AA JEV core were generated from WT or IPO7-KO Huh7. These SRIPs were infected Vero cells. Nluc reporter activity was quantified using the Nano-Glo Luciferase Assay System. **(D)** The core, prME, and replicon plasmids were transfected into WT or IPO7-KO Huh7. Intracellular SRIPs were collected after each repeat of freeze–thaw cycles. **(E)** *In vitro*-transcribed JEV replicon RNA was electroporated into WT and IPO7-KO Huh7 cells. Viral RNA 4 and 48 h post-transfection was calculated based on Nluc activity at 4 h post-electroporation. **(F)** SRIPs of Zika virus (ZIKV), dengue virus (DENV), or yellow fever virus (YFV) were generated from WT or IPO7-KO Huh7 cells. The reporter activity of the SRIPs was determined after infection into Vero cells. All data are presented as the mean ± SD of three independent experiments. Significance (**p < 0.01; n.s., not significant) was determined using Student’s *t*-test (*n* = 3; B-D and F, n = 4; E). Created with Biorender.com.

The above results suggest that IPO7-dependent nuclear translocation of flavivirus core proteins may play a role in virus release. To examine the importance of nuclear translocation in the viral life cycle, we employed single-round infectious particles (SRIPs) of flaviviruses [30,31]. Three kinds of plasmids, replicon, core, and prME expression plasmids, were transfected into packaging cells to produce SRIPs. The SRIPs obtained were used to infect Vero cells, and NanoLuc luciferase (Nluc) values of the SRIP-infected Vero cells were quantified (Fig 7A). SRIPs were generated using a yellow fever virus (YFV)-derived replicon and prME plasmid and WT JEV core or GP/AA JEV core plasmid. Nluc values in Vero cells infected with GP/AA JEV core SRIPs were significantly lower than those in cells infected with WT JEV core SRIPs (Fig 7B). This suggested that the GP/AA JEV core, which lacks nuclear transport of core proteins, was involved in the production of infectious virus particles. SRIPs harboring WT or GP/AA core were generated using WT and IPO7-KO Huh7 cells to further investigate the role of nuclear translocation on the production of infectious virus particles. Although the impaired production of SRIPs harboring WT core was observed in IPO7-KO Huh7 cells, the production of SRIPs harboring GP/AA core showed no difference between the WT and IPO7-KO Huh7 cells (Fig 7C). We examined the intracellular infectious virus and showed that the numbers of intracellular infectious viruses were equivalent between WT or IPO7-KO Huh7 cells (Fig 7D). Subsequently, we examined the involvement of IPO7 in viral RNA replication. *In vitro*-transcribed RNA was electroporated into WT or IPO7-KO Huh7 cells, and Nluc values were monitored. Consistent with the results shown in Fig 6B, Nluc values in IPO7-KO Huh7 cells were significantly higher than those in WT Huh7 cells (Fig 7E). We then evaluated the release of SRIP, which harbors core proteins of ZIKV, DENV, and YFV, in WT and IPO7-KO Huh7 cells. Similar results were obtained using DENV, ZIKV, and YFV SRIPs (Fig 7F). We checked the maturation of prM cleavage by furin. The expression of prM was slightly increased in JEV-infected IPO7-KO Huh7 cells (S3D Fig), suggesting nucleolar translocation of core protein might be partially involved in maturation of viral particles by furin cleavage. Our results suggest that IPO7-mediated core protein nuclear translocation is important for releasing infectious flavivirus particles.

## Discussion

This study aimed to elucidate the molecular mechanism underlying the nuclear translocation of flavivirus core proteins and the relevance of this translocation to the viral life cycle. We identified IPO7, also known as RanBP7, as a nuclear transporter of core proteins of flaviviruses such as DENV, ZIKA, WNV, and JEV. Although nuclear transport of WNV core protein was partially inhibited in IPO7-KO Huh7 cells, our results showed that IPO7 is involved in a nuclear transport of core proteins of *Flaviviridae* family. As the interaction between WNV core protein and importin-α/β enables WNV to efficiently produce viral particles [32], nuclear translocation of WNV core protein might be regulated by several nuclear carriers. IPO7 is an importin-β family member and can transport specific cargos, including ribosomal proteins such as L23a, S7, and L5 [29], histones [33,34], thyroid hormone receptor α1 (TRα1) [35], Smads [36,37], Erk [38], and YAP [39]. IPO7 has also been shown to transport histone H1 in a heterodimer with importin β1 into the nucleus [40,41]. Regarding viral infection and replication, IPO7 has been reported to regulate viral proteins, such as HIV-1 integrase [42] or purified intracellular reverse transcription complex [43], and inhibit virus replication. Using stable IPO7-knockdown cells, the nuclear entry of viral genomic DNA has been demonstrated to involve IPO7 [44], indicating the physiological significance of IPO7 in the life cycle of HIV-1. IPO7 is also stabilized upon Epstein-Barr Virus infection. IPO7 is required for the survival of host cells [45]. We found that IPO7-KO Huh7 cells showed impaired infectious virus production of flaviviruses. Similar results were obtained with GP/AA core protein (Fig 7B) and in a previous study [15], suggesting that core protein nuclear localization plays a role in viral release. In contrast, JEV RNA replication but not DENV and ZIKV was increased in IPO7-KO Huh7 cells. Further studies are required to determine whether IPO7 may transport other factors possessing antiviral activity against flaviviruses such as JEV.

There is growing evidence that the consensus NLS sequence recognized by IPO7 is enriched in basic amino acids (EKRKI(E/R)/(K/L/R/S/T)) [46]. Although no corresponding sequences are found in flavivirus core proteins, they harbor characteristic basic amino acid sequences, particularly in the C-terminus [47]. Indeed, the NLS of WNV is localized in the amino acid region 85–101 and is recognized by importin-α [32]. In DENV, three NLSs, ^6^KKAR^9^, ^73^KKSK^76^, and a bipartite NLS ^85^RKeigrmlnilnRRRR^100^ have been reported [48–50]. In contrast, in our study, IJ experiments showed no effect of Bimax, an importin-α inhibitor, on the nuclear entry of the JEV core protein [22]. Considering the cytoplasmic localization of all flavivirus core proteins assessed in IPO7-KO cells, we suggest that IPO7 serves as a common nuclear transporter for core proteins of flaviviruses.

While RNA viruses, including flaviviruses, are considered to replicate in the cytoplasm of mammalian cells, increasing evidence suggests nucleolar localization of the viral proteins [51]. Understanding the relationship between the nucleolar localization of core proteins and their function in virus replication is highly intriguing. Among the 20 importin-β family members, flaviviruses preferentially use IPO7 as a core protein nuclear transporter. There may be a physiological association in that IPO7 may transport several ribosomal proteins into the nucleus and may thus be involved in ribosomal biogenesis [29,52]. In addition, IPO7 has been shown to inefficiently release the ribosomal protein RPL23a, even in the presence of RanGTP, suggesting the presence of other molecules that can release the IPO7-ribosomal protein complex [29]. In our binding assay, the IPO7–RPS7 bond dissociated inefficiently despite the addition of excess amounts of RanGTP Q69L. It may be a candidate associating protein of nucleolar phosphoprotein B23, which we previously identified to bind with the JEV core protein [53]. Although it is unclear whether B23 is a target of core protein localizing in the nucleolus, interactome analysis may clarify why flavivirus core proteins are specifically recognized by IPO7 and transported into the nucleolus. DENV infections have recently been reported to induce the unfolded protein response, leading to the upregulation of spliced XBP1 (sXBP1), a transcriptional factor for protein folding. The complex of importin-α, importin-β, and sXBP1 is translocated to the nucleus to induce the transcription of genes encoding chaperones, which facilitates correct protein folding of NS1, a secretory viral protein [54]. The hepatitis C virus, belonging to hepacivirus, translocates nuclear pore complex (NPC) to double-membrane vesicles. Therefore, the Flaviviridae family may target nuclear transport machinery for efficient viral propagation, potentially providing a target for the development of antiviral drugs.

Alternatively, the nucleolar structure has also been considered to guide the nucleolar targeting of core proteins. We previously reported that several compounds, such as CDK inhibitors, drastically disrupted the nucleolar morphology and significantly suppressed viral replication [16]. These and our current data suggest that a functional correlation between the nucleolar structure and core protein might be required for proper capsid formation or envelopment and resulted in the production of infectious virus. The nucleolus is known to be generated by phase separation [55]. Furthermore, flavivirus capsid proteins show phase separation characteristics [56,57]. We assume that core protein nucleolar localization may occur via phase separation in the nucleolus. Further studies are required to better understand the physiological functions of flavivirus core proteins in the nucleus and nucleolus.

In summary, it is the first report identify that nuclear localization of core protein is mediated by IPO7 and plays an important role in the propagation of flaviviruses. These findings have contributed to further understand the flavivirus life cycle and its possibility as therapeutic target. However, the function of core protein in the nucleus and nucleolus is still unclear. Further work will be needed to unveil more details of physiological function of core proteins in the nucleus that lead to the efficient release of infectious viral particles. This approach might identify a novel drug target for flavivirus infectious diseases. Therefore, it is necessary to identify the condition to inhibit the nuclear localization of core proteins and evaluate its importance in vivo.

## Materials and methods

### Viruses and cells

293T (ATCC), Huh7 (JCRB0403, JCRB Cell Bank), and Vero (JCRB0111) cells were cultured in Dulbecco’s modified Eagle’s medium (DMEM, Wako) containing 10% fetal bovine serum (FBS) (Gibco) and penicillin/streptomycin (100 U/mL, Invitrogen) (P/S) at 37 °C in the presence of 5% CO_2_. C6/36 (IFO50010), a Singh’s *Aedes albopictus* cell clone, was maintained in Leibovitz’s L-15 medium (Thermo Fisher Scientific) supplemented with 10% FBS, 2.4 g/L tryptose phosphate broth (BD), and P/S. *Spodoptera frugiperda* (Sf-9) cells were maintained in Sf-900 II SFM medium (Thermo Fisher Scientific). All cell lines were routinely tested to be negative for mycoplasma contamination. JEV (strain AT31), DENV (strain 16688), and ZIKV (strain MR766) were obtained from National Institute of Infectious Diseases (NIID) and maintained in C6/36 cells. Infectious titers of JEV, DENV, and ZIKV were determined using the focus-forming unit (FFU) assay.

### Plasmid generation

cDNAs of the core proteins of JEV (strain AT31), DENV (strain 16681), and ZIKV (strain MR766) were PCR-amplified from cDNA from virus-infected cells, and cDNA of the WNV (strain NY99) core protein was PCR-amplified from cDNA synthesized at IDT DNA Technologies (Coralville, IA, USA). The cDNAs were cloned into pCAGGS or pFastBac-1 together with AcGFP and a flag-one-strep (FOS) tag. *IPO7* cDNA was PCR-amplified using primers 5′-GACCCAAGGAGGATCCATGGACCCCAACACC-3′, 5′-TCTAGAGTCGCGGCCGCTCAATTCATCCCTGG-3′ and cloned into pGEX6P-2 (Cytiva, WA, USA). The relevant oligonucleotide of SV40 large T antigen NLS (PPKKKRKVED) was ligated into the pGEX2T vector (GE Healthcare) carrying the *GFP* gene at the C-terminus of the multi-cloning site to produce GST-SV40NLS-GFP protein (referred to as SV40 NLS). Plasmids for GST-fused and flag-tagged importin-α1 (KPNA2), importin-β1, WT Ran, and Q69LRan were obtained as previously described [58–61]. RPL23A and RPS7 cDNAs were amplified from cDNA of Huh7 cells and cloned into pGEX6P-2. sgRNAs of human IPO7 (5′-ATGATCGACCTGAGTTACCA-3′), (5′-AATACATACCTGATGAGCTC-3′), and (5′-CCTGGTGCTGTTTCTCGATC-3′) were cloned into pX330. The plasmids for SRIPs have been previously reported [30]. The Nluc gene was inserted into pCMV JErep [62] designed as pCMV JErep nluc to generate the JEV replicon possessing a reporter gene. PCR was performed using pCMV JEVrep-nluc as a template and the following primers to obtain a replicon RNA using T7 promoter: forward primer 5’- CGGTACCCGGGGATCCTAATACGACTCACTATAAGAAGTTTATCTGTGTGAACTTCTT-3’ and reverse primer 5’-CGACTCTAGAGGATCCTAAGATACATTGATGAGTTTGGACAA-3‘. The PCR product was inserted into pMW119 and designated pMW119 T7 JEVrep-nluc. All cloning experiments were performed using an In-Fusion HD Cloning Kit (Takara Bio Inc., Shiga, Japan), and plasmid sequences were confirmed by DNA sequencing (at the Core Instrumentation Facility, Research Institute for Microbial Diseases, Osaka University, Osaka, Japan).

### Generation of recombinant JEV

The cDNA of JEV Nakayama strains was obtained from Tomohiro Ishikawa (Dokkyo Medical University), and the genome of JEV was divided into six fragments by PCR. Cloning was performed into pBlueScript SKII(+). The cytomegalovirus (CMV) promoter, hepatitis delta virus ribozyme (HDVr) site, and bovine growth hormone polyadenylation signal (BGH poly A) were synthesized by Integrated DNA Technologies (CA, USA) as a transcriptional cassette for JEV RNA and cloned into pBlueScript SKII(+). The fragment contained the core sequence with Gly42 and Pro43 mutated to Ala. Circular polymerase extension reaction products were synthesized as previously described [63] and transfected into Huh7 cells to generate recombinant JEV.

### Purification of recombinant proteins

Bacterially expressed recombinant proteins fused to GST were purified as previously described [64]. Importin-α and Ran proteins lacking GST were prepared by cleavage with PreScission protease (20 U/mg of fusion protein; GE Healthcare, Uppsala, Sweden) at 4°C for 12 h. The GST-cleaved Ran protein was equilibrated with buffer (50 mM HEPES [pH 7.3], 75 mM NaCl, 1 mM MgCl_2_, 1 mM dithiothreitol [DTT], and 0.1 mM phenylmethylsulfonyl fluoride) and incubated with GDP or GTP (2 mM; Sigma-Aldrich, St. Louis, MO, USA) and WT RanGDP or Q69LRanGTP on ice for 1 h. To stop the reaction, the proteins were incubated with 50 mM MgCl_2_. The proteins were equilibrated with dialysis buffer (20 mM HEPES [pH 7.3]), 100 mM CH_3_COOK, 2 mM DTT, and protease inhibitor (Protease Inhibitor Cocktail, Nacalai Tesque, Kyoto, Japan) using an Amicon Ultra device (10K; Millipore, Billerica, MA, USA).

### Generation of recombinant core proteins using an insect expression system

Recombinant baculoviruses (AcNPV) were generated using pFastBac1 AcGFP-JEVcore-FOS and a Bac-to-Bac system (Invitrogen, Carlsbad, CA, USA) following the manufacturer’s protocol. Recombinant baculoviruses carrying JEV core protein (JEVcore) cDNA were propagated in Sf-9 cells. To generate recombinant JEV core protein, Sf-9 cells (2 × 10^6^) were seeded in 10-cm dishes (Greiner Bio-One GmbH, Frickenhausen, Germany) and were infected with recombinant AcNPV at a multiplicity of infection of 10 and incubated for 3 days. Cells were collected and lysed in lysis buffer (20 mM Tris-HCl [pH 7.4], 135 mM NaCl, 1% Triton X-100, 1% glycerol, and protease inhibitor cocktail tablets [Roche Diagnostics, Mannheim, Germany]) at 4°C for 30 min, followed by centrifugation at 14,000 × *g* at 4°C for 15 min. The supernatants were incubated with Strep-Tactin Sepharose (IBA GMBH) overnight. The beads were washed using Strep-tag washing buffer (IBA GMBH) three times and eluted using elution buffer (IBA GMBH) containing D-desthiobiotin. The recombinant proteins were concentrated using an Amicon Ultra (10K; Millipore, Billerica, MA, USA).

### Microinjection

Huh7 cells were seeded on coverslips (MATSUNAMI) in 35-mm dishes (Corning, Acton, MA, USA). Within 1 min, AcGFP-JEVcore recombinant protein and Alexa Fluor 594 or 555-conjugated antibody (2 mg/mL, Thermo Fisher Scientific, as an injection marker) were microinjected into the cytoplasm at a final concentration of 1 mg/mL. After incubation for 30 min, the cells were fixed with 3.7% formaldehyde in PBS.

### Semi-intact nuclear import assay

Huh7 cells were treated with 40 μg/mL digitonin (Nacalai Tesque, Kyoto, JAPAN)) on ice for 5 min and washed twice with the transport buffer (20 mM HEPES [pH 7.3], 110 mM CH_3_COOK, 5 mM CH_3_COONa, 2 mM (CH_3_COO)_2_Mg, 0.5 mM EGTA, 2 mM DTT, and protease inhibitor [Nacalai Tesque]) to minimize residual protein content in the cytoplasm. The permeabilized cells were incubated in ice-cold transport buffer for 10 min and then incubated at 37°C for 30 min in the following transport mixtures: for the experiment shown in Fig 1C, 4 pmol of SV40 NLS protein, 20 pmol of AcGFP-JEVCoreWT and AcGFP-JEVCoreGP/AA or 10 pmol of GST-GFP-RPL23A, Rabbit Reticulocyte Lysate (Promega), ATP regeneration system (0.5 mM ATP [Wako]), 20 U/mL creatine phosphokinase (Sigma-Aldrich), and 5 mM creatine phosphate (Sigma-Aldrich); for the experiment shown in Fig 4C, 4 pmol of IPO7 and 40 pmol of WT RanGDP with ATP regeneration system in a total volume of 10 μL per sample. After incubation, the cells were fixed with 3.7% formaldehyde in PBS. The cells were observed under a Nikon Eclipse 80i microscope (Nikon, Tokyo, Japan).

### Immunofluorescence

Cells were fixed with 3.7% formaldehyde in PBS at 25°C for 15 min. After washing in PBS, the cells were treated with 0.1% Triton X-100 in PBS for 5 min, blocked in PBS containing 5% skim milk for 30 min, and incubated in appropriately diluted (in PBS containing 5% skim milk) primary antibodies at room temperature for 2 h or at 4°C overnight. Subsequently, the cells were incubated with Alexa Fluor-conjugated secondary antibodies (fluorescence at 488, 594, 555, or 645 nm; Thermo Fisher Scientific, Rockford, IL, USA). Nuclei were counterstained with DAPI (1:10,000 in PBS; Dojindo Laboratories, Kumamoto, Japan) for 10 min. The samples were examined using a Zeiss Axiophot fluorescence microscope (Carl Zeiss, Gottingen, Germany) and a Nikon Eclipse 80i microscope (Nikon).

### Antibodies

The following antibodies were used in this study: mouse monoclonal antibodies against JEV/DENV/ZIKV NS3 (clone 578) [65], anti-IPO7 (ab99273; Abcam, Cambridge, UK), anti-JEV core (GTX131368; GeneTex, Irvine, CA, USA), anti-flag monoclonal (clone M2; Sigma, St. Louis, MO, USA), anti-Strep-tagII (4F1, MBL, Tokyo, JAPAN), anti-GFP (JL-8, Takara, Shiga, JAPAN), anti-HA (3F10, Roche, Mannheim, Germany), anti-DENV core (GTX633632; Genetex, Irvine, CA, USA), anti-DENV prM (GTX134828; Genetex, Irvine, CA, USA) antibodies.

### Virus infection

Huh7 cells were seeded in 24-well plates at 3 × 10^4^ cells/well and incubated for 24 h. Subsequently, the cells were infected with JEV, DENV, or ZIKV at a multiplicity of infection (MOI) of 5 for 2 h. The virus-infected Huh7 cells were washed with PBS and cultured in culture medium. Viral infectivity was determined as FFUs using an immunostaining assay. Viability of virus-infected cells was determined in previous reports [65].

### FFU assay

The FFU assay was performed as previously described [14]. Culture media from Huh7 cells infected with JEV were collected and serially diluted. The diluted media including viruses were added to Vero cells seeded in 24-well plates (5 × 10^4^ cells/well). After 2 h of adsorption, the cells were washed with PBS followed by culture in DMEM containing 10% FBS, 100 U/mL penicillin and 100 μg/mL streptomycin, and 1.25% methylcellulose 4,000cP (M0512, Merck KGaA, Darmstadt, Germany). Two days later, the cells were fixed with 4% paraformaldehyde and permeabilized with 0.5% Triton X-100 for 5 min. The cells were incubated with an anti-NS3 (clone 578) antibody [65] for 30 min and then with a biotin-conjugated anti-mouse IgG secondary antibody (Vector Laboratories, Burlingame, CA, USA) for 30 min. After washing with PBS, infectious foci were visualized by incubation with Streptavidin Biotin Complex Peroxidase (Nacalai Tesque) for 30 min followed by incubation with VIP Peroxidase Substrate (Vector Laboratories) for 10 min.

### Western blotting

Cell lysates were prepared by incubation in Onyx lysis buffer (20 mM Tris-HCl [pH 7.4], 135 mM NaCl, 1% Triton X-100, 1% glycerol, and protease inhibitor cocktail tablets [Roche Molecular Biochemicals]) at 4°C for 15 min. After sonication, the lysates were centrifuged at 13,000 x g at 4°C for 5 min. Protein concentrations were determined using Protein Assay Dye Reagent Concentrate (Bio-Rad). Supernatants were incubated with sample buffer at 95°C for 5 min. The samples (50 μg of proteins) were resolved by SDS-PAGE (Novex/Life Technologies) and transferred onto nitrocellulose membranes (iBlot/Life Technologies). The membranes were blocked with PBS containing 5% skim milk and incubated with primary antibodies at 4°C overnight. After washing, the membranes were reacted with HRP-conjugated secondary antibody at room temperature for 2 h. The immune complexes were visualized using Super Signal West Femto substrate (Pierce) and detected using an Amersham ImageQuant 800 system (Cytiva Life Science, Marlborough, MA, USA).

### MS

293T cells transfected with pCAG AcGFP-FOS or pCAG AcGFP-JEVcore-FOS were incubated for 48 h and lysed in lysis buffer (20 mM Tris-HCl [pH = 7.4], 135 mM NaCl, 1% Triton X-100, 1% glycerol, and protease inhibitor cocktail tablets [Roche]). The cell lysates were incubated with Strep-Tactin Sepharose (IBM GmbH) for 90 min and washed three times with lysis buffer. The precipitates were boiled in sample buffer at 95°C for 5 min as previously described. The proteins were subjected to SDS-PAGE, stained with CBB, purified, and divided into 10 aliquots. The proteins were reduced with 10 mM DTT, alkylated with 55 mM iodoacetamide, and digested with trypsin (Promega). The peptides were subjected to MS using an LC-ESI system. MS data were obtained by nanocapillary reversed-phase LC-MS/MS using a C18 column (0.1 × 150 mm) on a nanoLC system (Advance/Michrom BioResources) coupled to an LTQ Orbitrap Velos mass spectrometer (Thermo Fisher Scientific). The mobile phase consisted of water containing 0.1% formic acid (solvent A) and acetonitrile (solvent B). Peptides were eluted using a gradient of 5–35% B for 45 min at a flow rate of 500 nL/min. The mass scanning range of the instrument was set to m/z 350–1,500. The ion spray voltage was set to 1.8 kV in the positive ion mode. The MS/MS spectra were acquired by automatic switching between MS and MS/MS modes (with collisional energy set to 35%). The dynamic exclusion was set to 30 s, with 10 ppm tolerance. Helium gas was used as a collision gas. The data obtained were processed using Proteome Discoverer (Thermo Fisher Scientific), and peptides were identified using MASCOT (Matrix Science, UK) against the Swiss-Prot human database. Precursor mass tolerance was set to 10 ppm, and MS/MS tolerance was 0.8 Da for Orbitrap and linear ion trap, respectively. Carbamidomethylation of cysteine was set as a fixed modification. Oxidation of methionine and N-terminal Gln to pyro-Glu was set as variable modifications. We used a Mascot peptide significance threshold of *p* < 0.05 for post-search filtering. Quantitative and fold exchange values were calculated using Scaffold4 (Proteome Software, USA) for MS/MS-based proteomic studies.

### Quantitative reverse transcription (RT-q)PCR

RNA was extracted using ISOGEN II (Nippon Gene, Tokyo, Japan), and JEV RNA was quantified using a Power SYBR green RNA-to-Ct 1-Step kit (Thermo Fisher Scientific, Waltham, MA, USA) and the AriaMx Real-Time PCR system (Agilent, Santa Clara, CA, USA). The primers used were JEV forward 5′- AGCTGGGCCTTCTGGT’ and reverse 5′- CCCAAGCATCAGCACAAG’, and β-actin forward 5′-TTGCTGACAGGATGCAGAAG-3′ and reverse 5′-GTACTTGCGCTCAGGAGGAG- 3′.

### Production of SRIPs

To generate JEV SRIPs, YFV-derived replicon plasmid (2.5 μg) [31], PrME plasmid (1.25 μg), and JEV core plasmid (1.25 μg) were transfected into Huh7 WT or IPO7 KO cells. Each culture medium, including SRIPs, was harvested three days post-transfection. SRIPs for ZIKV, DENV, and YFV were generated using replicon, PrME, and core plasmid for each virus. Infectious titers were evaluated by the reporter activity on Vero cells infected with SRIPs one day post-infection using the Nano-Glo Luciferase Assay System and the GloMax Discover plate reader (Promega, WI, USA).

### Evaluation of viral replication using replicon RNA

pMW119 JEVrep-nluc was digested using SalI. Replicon RNA was synthesized using the mMESSAGE mMACHINE T7 Ultra Kit (Thermo Fisher Scientific, Waltham, MA, USA). Briefly, 10 μg of the transcribed RNA was electroporated at 270 V and 960 μF by a Gene Pulser (Bio-Rad, Hercules, CA) into 10 million cells per mL, suspended in 25 mL of culture medium, and seeded at a volume of 1 mL per well in 12-well culture plates. Nluc activity was measured at 4 and 48 h post-transfection using a Nano-Glo Luciferase Assay System (Promega, WI, USA) following the manufacturer’s protocol. The relative luciferase activity is presented as the ratio of the Nluc activity measured at 48 h post-transfection to that at 4 h.

### Statistical analysis

Normally distributed data (as determined using NORMDIST in Excel (Microsoft, version 16.75) were analyzed using two-tailed, unpaired parametric *t*-tests. Nonparametric data were analyzed using the Mann–Whitney U test or Kruskal–Wallis test followed by Dunn’s multiple comparison tests. Statistical analysis was performed using the GraphPad PRISM software (version 9.0.0, GraphPad Software, MA, USA*)*. Proteins were quantified via WB, and the ratio of nuclear transported proteins was calculated using the Image J software (https://imagej.net/ij/index.html, 1.54g). Significance was set to *p* < 0.05. Experimental schemes were prepared using BioRender.

## Supporting information

S1 Fig Purified recombinant proteins were subjected to SDS-PAGE, and immunoblotting was performed using anti-GFP or anti-Strep tag antibodies.
(TIFF)

S2 Fig **(A, B)** IPO7 expression in IPO7-KO Huh7 cell clones was confirmed by western blotting using the anti-IPO7 antibody. **(C)** Plasmids encoding GFP fused with core proteins of *Flaviviridae* were transfected into WT or IPO7-KO Huh7 cells. The scale bar indicates 20 μm. **(D)** The plasmid encoding HA-tagged DENV NS5 was transfected into WT or IPO7-KO Huh7 cells. NS5 localization was detected using the anti-HA antibody. The scale bar indicates 20 μm. The right graph is the quantification of the ratio of nuclear translocation. **(E)** The WT or IPO7-KO Huh7 cells were infected with DENV. Subcellular localization of NS5 protein was observed using indicated antibodies. The scale bar indicates 20 μm. The right graph is the quantification of the ratio of nuclear translocation. Data are presented as the mean ± SD of three independent experiments.
(TIFF)

S3 Fig **(A)** WT Huh7 or four IPO7-KO Huh7 cell clones were infected with JEV. At 2 dpi, viral titers in supernatants were determined. **(B)** IPO7 expression in IPO7-KO 293T cells was confirmed through western blotting. **(C)** WT or IPO7-KO 293T cells were infected with JEV. At two dpi, viral titers in supernatants were determined by the FFU assay. **(D)** DENV was infected in WT or IPO7-KO Huh7 cells. After two days, mock or virus-infected cells were subjected to SDS-PAGE and western blotting using indicated antibodies. Data are presented as the mean ± SD of three independent experiments. Significance (**p < 0.01) was determined using Student’s *t*-test (*n* = 3).
(TIFF)
