# Supplementary figures and images for: Importin-7-dependent nuclear translocation of the Flavivirus core protein is required for infectious virus production
Source: PLoS Pathog. 2024 Aug 15;20(8):e1012409. doi: 10.1371/journal.ppat.1012409 (PMC11326614; doi:10.1371/journal.ppat.1012409)

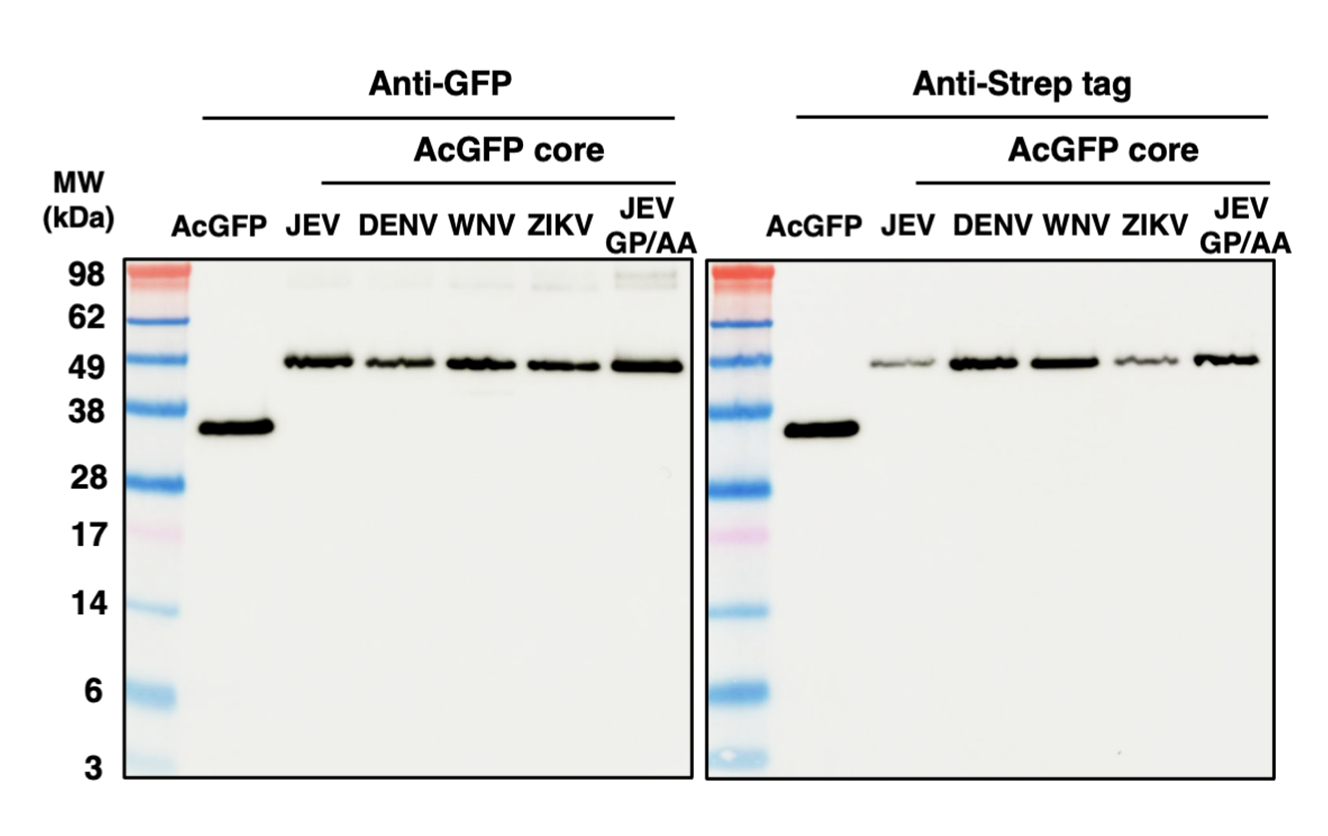

Supplement: S1 Fig — Purified recombinant proteins were subjected to SDS-PAGE, and immunoblotting was performed using anti-GFP or anti-Strep tag antibodies. (TIFF) [file ppat.1012409.s001.tiff]

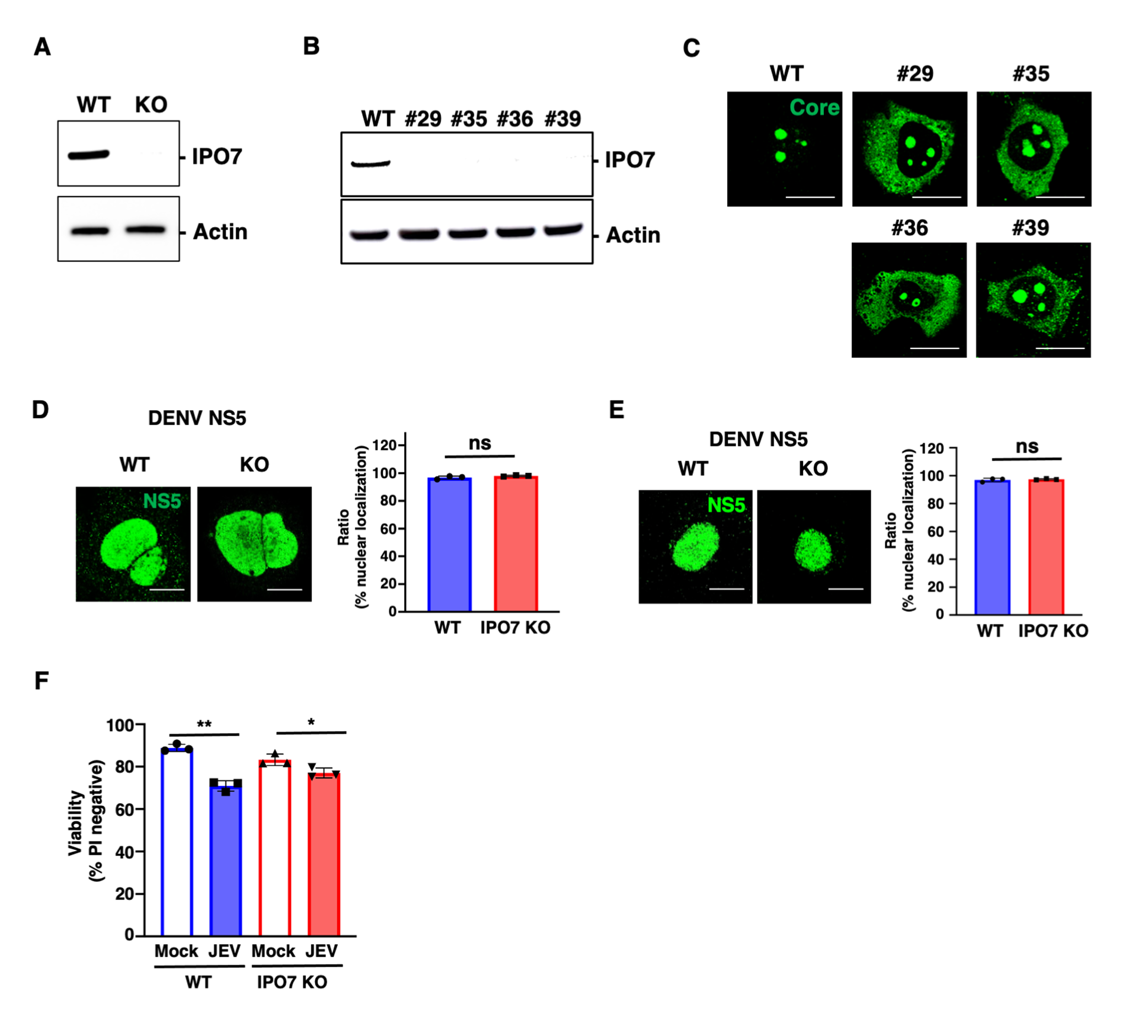

Supplement: S2 Fig — (A, B) IPO7 expression in IPO7-KO Huh7 cell clones was confirmed by western blotting using the anti-IPO7 antibody. (C) Plasmids encoding GFP fused with core proteins of Flaviviridae were transfected into WT or IPO7-KO Huh7 cells. The scale bar indicates 20 μm. (D) The plasmid encoding HA-tagged DENV NS5 was transfected into WT or IPO7-KO Huh7 cells. NS5 localization was detected using the anti-HA antibody. The scale bar indicates 20 μm. The right graph is the quantification of the ratio of nuclear translocation. (E) The WT or IPO7-KO Huh7 cells were infected with DENV. Subcellular localization of NS5 protein was observed using indicated antibodies. The scale bar indicates 20 μm. The right graph is the quantification of the ratio of nuclear translocation. Data are presented as the mean ± SD of three independent experiments. (TIFF) [file ppat.1012409.s002.tiff]

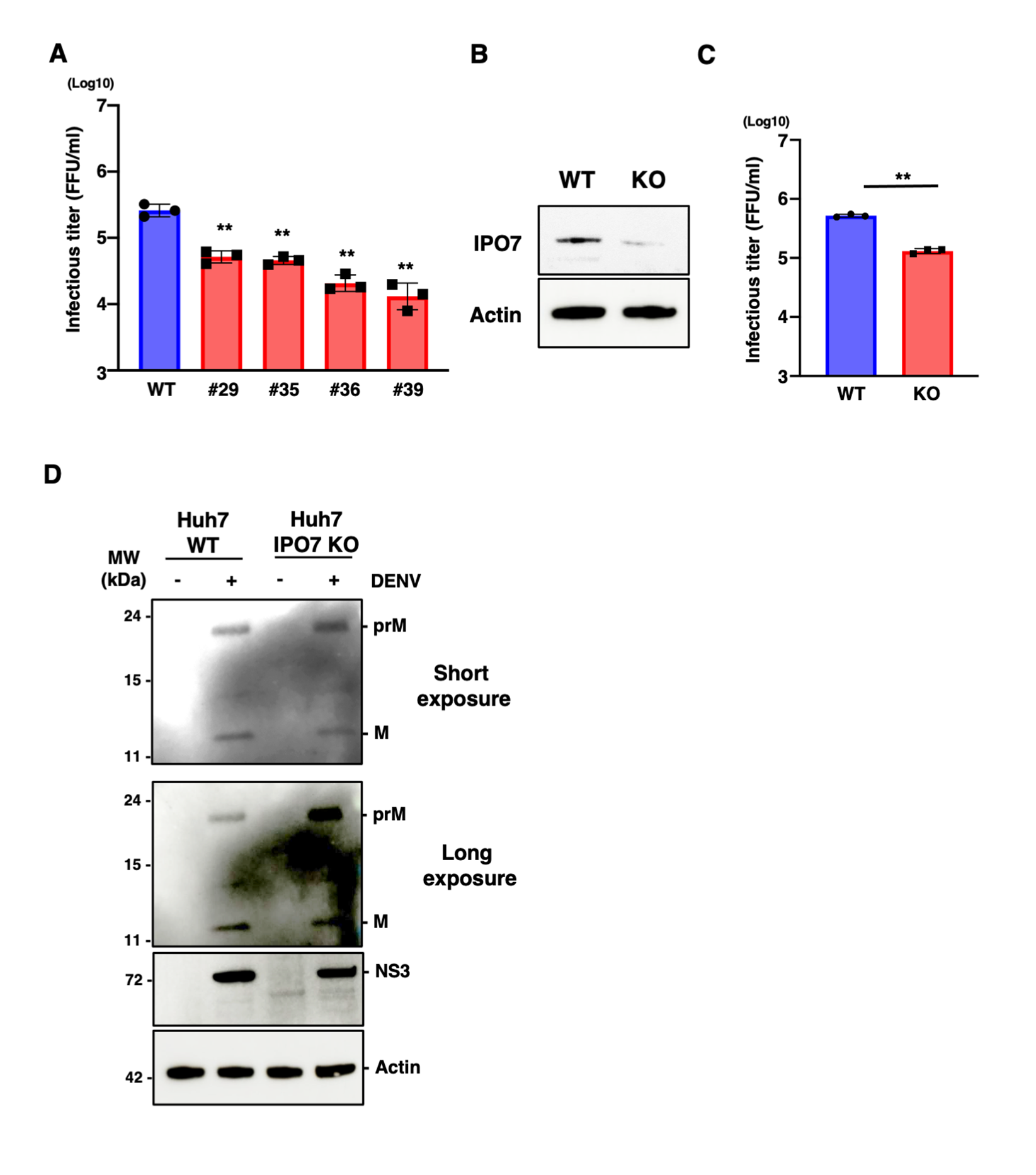

Supplement: S3 Fig — (A) WT Huh7 or four IPO7-KO Huh7 cell clones were infected with JEV. At 2 dpi, viral titers in supernatants were determined. (B) IPO7 expression in IPO7-KO 293T cells was confirmed through western blotting. (C) WT or IPO7-KO 293T cells were infected with JEV. At two dpi, viral titers in supernatants were determined by the FFU assay. (D) DENV was infected in WT or IPO7-KO Huh7 cells. After two days, mock or virus-infected cells were subjected to SDS-PAGE and western blotting using indicated antibodies. Data are presented as the mean ± SD of three independent experiments. Significance (**p < 0.01) was determined using Student’s t-test (n = 3). (TIFF) [file ppat.1012409.s003.tiff]
